# Feature and impact of guideline-directed medication prescriptions for heart failure with reduced ejection fraction accompanied by chronic kidney disease

**DOI:** 10.7150/ijms.55119

**Published:** 2021-04-28

**Authors:** Yung-Lung Chen, Chi-Ling Hang, Chien-Hao Su, Po-Jui Wu, Huang-Chung Chen, Hsiu-Yu Fang, Yen-Nan Fang, Cheng-I Cheng, Morgan Fu, Shyh-Ming Chen

**Affiliations:** 1 Section of Cardiology, Department of Internal Medicine, Kaohsiung Chang Gung Memorial Hospital, Kaohsiung City, Taiwan, Republic of China.; 2 Chang Gung University College of Medicine, Taoyuan City, Taiwan, Republic of China.; 3 Department of Pharmacy, Kaohsiung Chang Gung Memorial Hospital, Kaohsiung City, Taiwan, Republic of China.

**Keywords:** chronic kidney disease, guideline-directed medications, heart failure with reduced ejection fraction, mortality

## Abstract

**Background:** With respect to total mortality and cardiovascular mortality, the feature and impact of guideline-directed medication (GDM) prescriptions for heart failure with reduced ejection fraction (HFrEF) with chronic kidney disease (CKD) are unknown. Therefore, we aimed to determine these aspects.

**Methods:** GDM prescriptions and their impact on discharged patients with and without CKD were analyzed. To analyze differences in one-year clinical outcomes, propensity score matching was conducted on a cohort of patients with concomitant HFrEF and CKD who received more and fewer GDM prescriptions.

**Results:** A total of 1509 patients were enrolled in Taiwan's HFrEF registry from May 2013 to October 2014, and 1275 discharged patients with complete one-year follow-up were further analyzed. Of these patients, 468 (36.7%) had moderate CKD, whereas 249 (19.5%) had advanced CKD. Patients with advanced CKD received fewer prescribed GDMs than other patients. Multivariate analysis revealed that peripheral arterial occlusive disease, thyroid disorder, advanced HF at discharge, diastolic blood pressure, digoxin use, and fewer prescribed GDMs were independent predictors of one-year total mortality. After propensity score matching, patients with fewer prescribed GDMs had higher one-year total mortality rate than those with more prescribed GDMs (*P*=0.036).

**Conclusions:** CKD at discharge from HF hospitalization was associated with fewer GDM prescriptions, particularly in patients with more advanced CKD. The propensity-matched analysis indicated that more GDM prescriptions led to better clinical outcomes in HFrEF patients with CKD. Careful interpretation of changes in renal function during HF hospitalization may improve GDM prescriptions.

## Introduction

Clinical guidelines recommend the use of medications such as angiotensin-converting enzyme inhibitors (ACEIs), angiotensin II receptor blockers (ARBs), angiotensin receptor-neprilysin inhibitors (ARNi), beta-blockers, and mineralocorticoid receptor antagonists (MRAs) to reduce adverse outcomes in patients with heart failure with reduced ejection fraction (HFrEF) 1, 2. Furthermore, the use of guideline-directed medications (GDMs) is very crucial to the clinical outcomes of patients with HFrEF 3.

Chronic kidney disease (CKD), one of the most common and important comorbidities in patients with HFrEF, is associated with worse clinical outcomes 4. Owing to concerns about hypotension, renal dysfunction, and hyperkalemia, patients with moderate and advanced CKD are less likely to receive GDM therapy 5.

The Taiwan Society of Cardiology (TSOC)-HFrEF Registry is a prospective, multicenter, observational survey of patients presenting to 21 medical centers in Taiwan. A previous report from the TSOC-HFrEF Registry indicated that the prescription rate at discharge was 62.1% for ACEIs or ARBs, 59.6% for beta-blockers, and 49.0% for MRAs 6. The lower GDM prescriptions in this prospective cohort registry may be related to CKD. Therefore, the present study aimed to investigate the feature and impact of GDM prescriptions in HFrEF patients with CKD with respect to total mortality and cardiovascular (CV) mortality. We hypothesized that misinterpretation of renal function during HF hospitalization could lead to inappropriate discontinuation of GDMs and that HF patients with CKD at discharge may receive fewer GDMs and have worse clinical outcomes than those receiving more GDMs.

## Materials and Methods

### Study design and patients

This present study is an observational, noninterventional prospective cohort study that retrieves data from the TSOC-HFrEF Registry. The study subjects were hospitalized patients who presented with either acute new-onset HF or acute decompensation of chronic HF with reduced left ventricular ejection fraction (LVEF <40%) and who were enrolled in the TSOC-HFrEF Registry. There were no specific exclusion criteria, except for patients aged <18 years. Data were collected after the patients provided signed informed consent. Patient data were collected during index hospitalization, starting from the initial point of care and ending with discharge or death. Data on follow-up status were collected at the 6^th^ and 12^th^ months.

**Figure 1:**
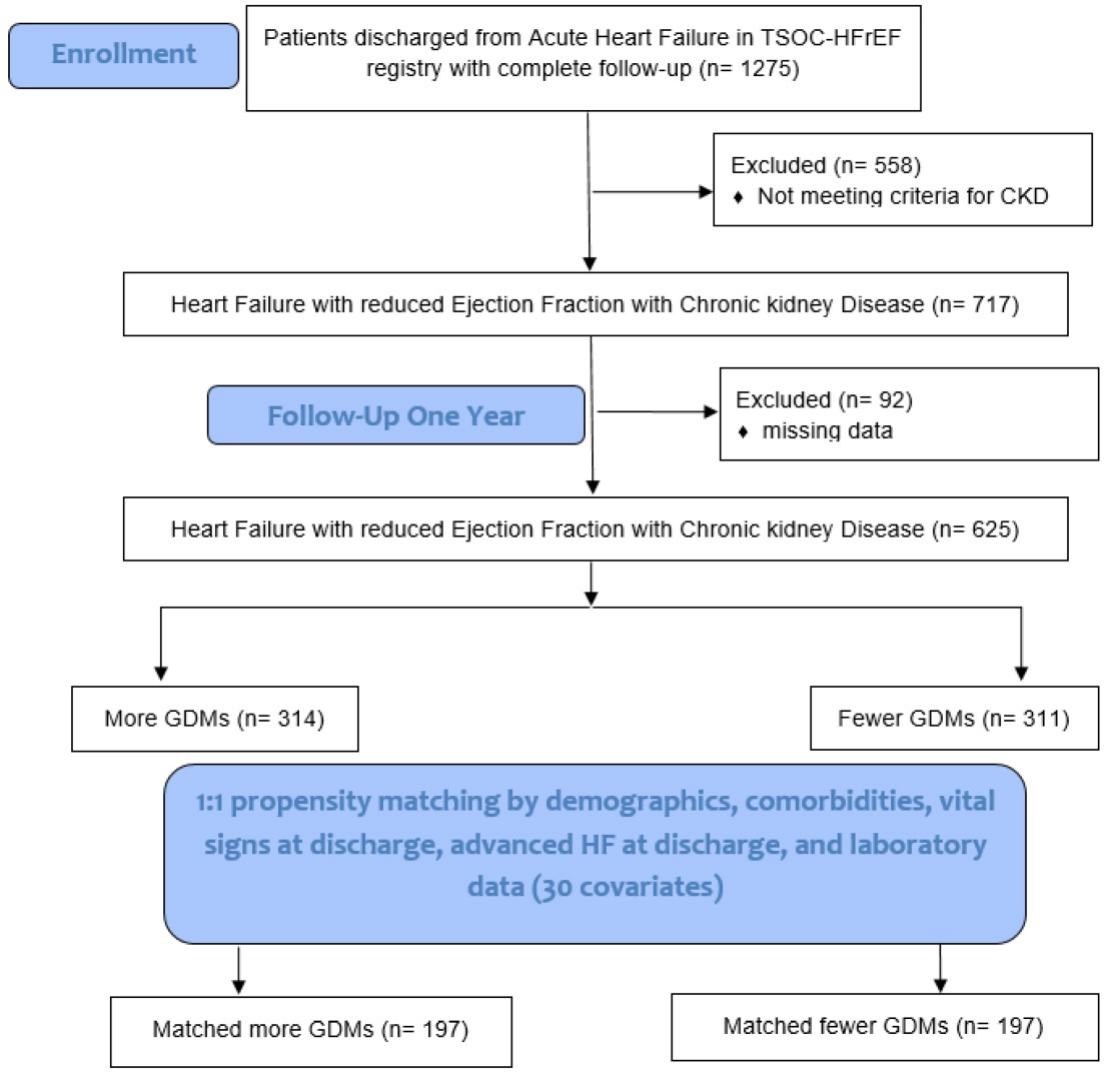
Study flowchart. TSOC-HFrEF registry: Taiwan Society of Cardiology Heart Failure with reduced Ejection Fraction registry. CKD: chronic kidney disease. GDM: guideline-directed medications. HF: heart failure.

We compared the feature of patients' characteristics and clinical outcomes among those HF patients with and without CKD. We evaluated the clinical predictors in terms of total mortality and CV mortality in HF patients with CKD. A propensity score was used to match HF patients with CKD with more GDMs and fewer GDMs to a 1:1 ratio by demographical and clinical covariates (Fig. 1). The Institutional Review Board of each center (102-1822B) approved the use of the registry and the study design. The detailed study protocol was described in a previous report 7.

### Definition

For this study, CKD was defined as an estimated glomerular filtration rate (eGFR) of <60 mL/min/1.73 m^2^ at discharge during index hospitalization. Patients in the registry were further divided into moderate CKD, advanced CKD, and control groups. The moderate CKD group comprised patients with eGFR between 60 and 30 mL/min/1.73 m^2^, whereas the advanced CKD group consisted of patients with eGFR of <30 mL/min/1.73 m^2^. All other patients were allocated to the control group (eGFR ≥60 mL/min/1.73 m^2^). The eGFR was calculated using the abbreviated Modification of Diet in Renal Disease study equation: eGFR (mL/min/1.73 m^2^) = 186.3 × (serum creatinine [mg/dL])^-1.154^ × (age [years])^-0.203^ × (0.742 if a woman) 8. Advanced HF was defined as HF with New York Heart Association (NYHA) functional class ≥3.

Prescribed GDM referred to the prescription of renin-angiotensin system blockers (ACEIs or ARBs), beta-blockers, or MRAs (spironolactone or eplerenone) according to clinical guidelines (Class I, Level A) 1,2. The prescription of any one of these three categories of medications was counted as one kind of GDM use. The concomitant prescription of ACEIs and ARBs was also counted as one kind of GDM use. More GDM prescriptions were defined as prescriptions of two kinds or more than two kinds (≥2) of GDMs, whereas fewer GDM prescriptions were defined as prescriptions of fewer than two kinds (<2) of GDMs. Prescriptions of ARNi (sacubitril/valsartan) and ivabradine were not included for analysis because these drugs were not approved for use in Taiwan and also not covered by Taiwan's National Health Insurance during the TSOC-HFrEF Registry period.

### Statistical analysis

Descriptive summaries are presented for all patients and for patient subgroups. Quantitative data are expressed as mean ± standard deviation, and categorical variables are reported as percentages. Student's *t*-test was employed for comparisons between continuous data, and the chi-square test or Fisher's exact test was utilized for comparisons between categorical data. A multivariate Cox proportional hazards model was used to analyze independent predictors of one-year total mortality and CV mortality. A 1:1 PSM between patients with concomitant HFrEF and CKD who received more GDM prescriptions and those who had fewer GDM prescriptions was conducted. Nearest-neighbor matching with a caliper size of 0.2 was performed to mitigate the effects of potential selection bias and reduce any imbalance in baseline patient characteristics, including demographics (age, sex, smoking, alcoholism, and body mass index [BMI]), comorbidities (diabetes mellitus [DM], old myocardial infarction, ischemic cardiomyopathy [ICM], admission due to acute decompensated HF, peripheral arterial occlusive disease [PAOD], chronic obstructive pulmonary disease [COPD], obstructive sleep apnea, thyroid disorder, depression, and cancer), echocardiographic parameters (LVEF), vital signs at discharge (systolic blood pressure [SBP] and diastolic blood pressure [DBP]), advanced HF at discharge, and laboratory data (serum sodium, potassium, and blood hemoglobin).

Event-free survival relative to total mortality and CV mortality before and after PSM in patients with concomitant HFrEF and CKD who received more and fewer GDM prescriptions was calculated using the Kaplan-Meier method and compared using the log-rank test. Cox regression analyses adjusted all covariates used to generate propensity score were performed to assess the association between GDM prescriptions and clinical outcomes in HFrEF patients with CKD. A *P*-value of <0.05 was considered to indicate statistical significance. Statistical analyses were performed using SPSS Statistics for Windows version 17.0 (SPSS Inc., Chicago, IL, USA) and NCSS version 12 (NCSS Statistical Software, Kaysville, UT, USA).

## Results

### Baseline characteristics, GDM prescriptions, and one-year total mortality of all enrolled HFrEF patients with and without CKD

A total of 1509 patients from 21 medical centers were enrolled in the TSOC-HFrEF Registry from May 2013 to October 2014. Detailed baseline characteristics are presented in our registry report 7. Of these patients, 36 (2.4%) died during index hospitalization, whereas 198 (13.2%) from the initial enrolling hospital were lost to follow-up. Overall, 1275 regularly followed-up patients were included for further analysis, 249 (19.5%) and 468 (36.7%) of whom had advanced CKD and moderate CKD, respectively.

Table 1 presents the differences among patients with advanced CKD, those with moderate CKD, and control subjects. Briefly, patients with CKD were significantly older, were more likely to be female, and had higher rates of DM, PAOD, ICM, and advanced HF at discharge than those without CKD (all *P*<0.001). Additionally, patients with CKD had higher blood urea nitrogen (BUN), creatinine, and potassium levels and lower sodium and hemoglobin levels (all *P*<0.001). SBP at discharge was higher in patients with CKD than in those without CKD (*P*<0.001). Moreover, fewer patients with CKD received ACEIs/ARBs and MRAs than those without CKD (*P*<0.001).

Patients with advanced CKD were more likely to be female and to have more DM, PAOD, ICM, and advanced HF at discharge than patients with moderate CKD and control subjects (all *P*<0.05). Patients with advanced CKD had higher BUN, creatinine, and potassium levels and lower sodium and hemoglobin levels (all *P*<0.05). SBP at discharge was higher in patients with advanced CKD than in those with moderate CKD (*P*<0.05). Furthermore, fewer patients with advanced CKD received ACEIs/ARBs, MRAs, and diuretics than those with moderate CKD (all *P*<0.05). Patients with advanced CKD had lower BMI and higher LVEF than patients with moderate CKD and control subjects (both *P*<0.05). The prescription rates for calcium channel blockers and antiplatelet medications were higher and the prescription rates for digoxin and anticoagulant medications were lower in patients with advanced CKD than in patients with moderate CKD and control subjects (all *P*<0.05). Most importantly, patients with advanced CKD had the lowest number of GDM prescriptions, whereas patients without CKD had the highest number of GDM prescriptions (1.1 ± 0.8 vs. 1.7 ± 0.9 vs. 1.9 ± 0.9, *P*<0.001). Patients with advanced CKD and moderate CKD had higher incidences of one-year total mortality and CV mortality than control subjects (detailed data are presented in Table 1).

### Baseline characteristics of patients with concomitant HFrEF and CKD with and without one-year total mortality and CV mortality

**Figure 2:**
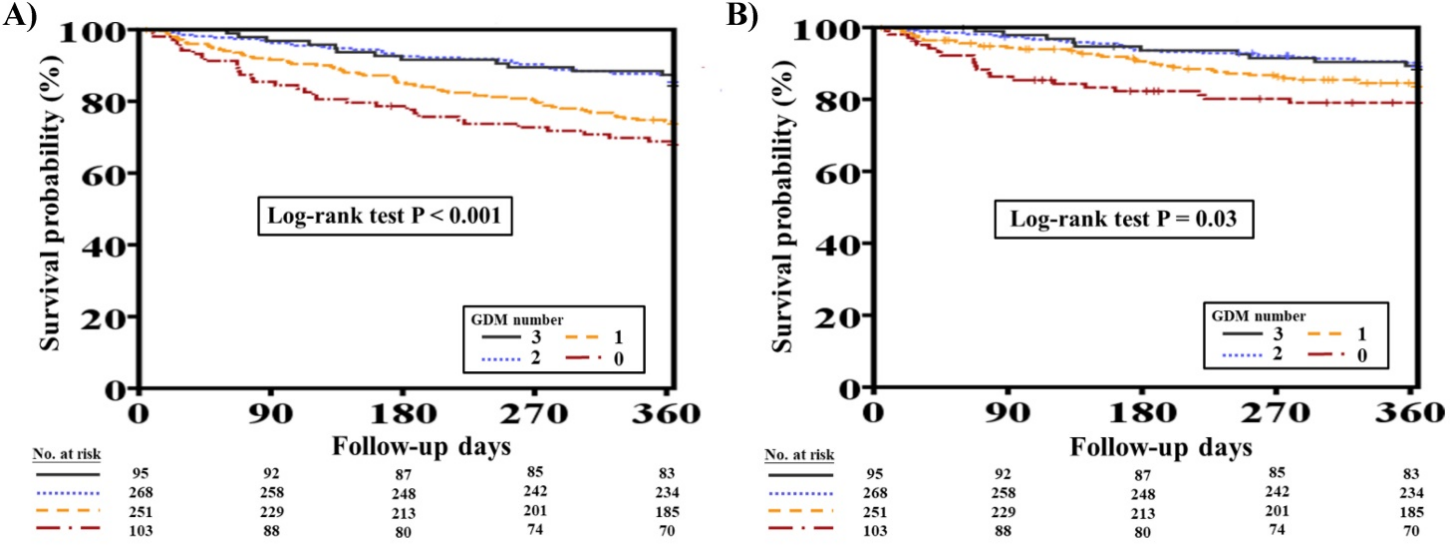
One-year total mortality and CV mortality rates in patients with concomitant HFrEF and CKD with different numbers of GDM prescriptions. **A,** Different numbers of GDM prescriptions were related to different one-year total mortality rates (log-rank test, *P*<0.0001). **B,** Different numbers of GDM prescriptions were related to different one-year CV mortality rates (log-rank test, *P*=0.03). CKD, chronic kidney disease; CV, cardiovascular; GDM, guideline-directed medication; HFrEF, heart failure with reduced ejection fraction.

Overall, 153 (21.3%) out of the original 717 patients with concomitant HFrEF and CKD died at one-year follow-up. Patients who died were significantly older (70.3 ± 12.1 vs. 66.7 ± 14.7 years, *P*=0.008) and were more likely to have had lower BMI, DM, advanced CKD, PAOD, COPD, thyroid disorder, and previous valvular surgery than patients who survived (all *P*<0.05). Furthermore, the deceased patients were more likely to have had lower hemoglobin levels during hospitalization (*P*<0.05). Additionally, patients who died at one-year follow-up had lower DBP, had more advanced HF at discharge, and received fewer prescriptions of ACEIs/ARBs and beta-blockers but more prescriptions of digoxin (all *P*<0.05). The number of GDM prescriptions was significantly higher among survivors than among deceased patients (1.6 ± 0.9 vs. 1.2 ± 0.9, *P*<0.001) (Supplementary Table 1). Figure 2A shows the clinical outcomes with respect to one-year total mortality in patients with different numbers of GDM prescriptions (log-rank test, *P*<0.001).

Of 717 patients with HFrEF and CKD, 100 (13.9%) suffered from CV mortality at one-year follow-up. Patients who suffered from CV mortality were more likely to have had hypertension, PAOD, thyroid disorder, and previous valvular surgery than those who survived (all *P*<0.05). The deceased patients were also more likely to have had higher BUN levels during hospitalization (*P*=0.004). Moreover, patients who had CV mortality at one year had lower SBP and DBP, had more advanced HF at discharge, and received fewer prescriptions of beta-blockers but more prescriptions of digoxin (all *P*<0.05). The number of GDM prescriptions was significantly lower among patients who had CV mortality at one year (1.3 ± 0.9 vs. 1.5 ± 0.9, *P*=0.007) (Supplementary Table 2). Figure 2B shows the clinical outcomes with respect to one-year CV mortality in patients with different numbers of GDM prescriptions (log-rank test, *P*=0.03).

### Multivariate analysis for predictors of one-year total mortality and CV mortality in HFrEF patients with CKD

Supplementary Table 1 summarizes all relevant variables, including demographics (age, sex, BMI), comorbidities (atrial fibrillation, DM, advanced CKD, PAOD, COPD, thyroid disorder), previous valvular surgery, HF type (new-onset HF or acute decompensation of chronic HF), HF etiology (ICM or non-ICM), laboratory data (sodium, potassium, and hemoglobin levels), echocardiographic data (LVEF), vital signs at discharge (DBP), advanced HF at discharge, and therapy (digoxin use, more GDM prescriptions). Multivariate analysis of all of these relevant variables revealed that PAOD (hazard ratio [HR]: 1.828, 95% confidence interval [CI]: 1.130-2.959; *P*=0.014), thyroid disorder (HR: 1.969, 95% CI: 1.101-3.521; *P*=0.022), advanced HF at discharge (HR: 1.688, 95% CI: 1.192-2.391; *P*=0.003), DBP at discharge (per mmHg decrement) (HR: 1.018, 95% CI: 1.004-1.033; *P*=0.014), digoxin use (HR: 1.563, 95% CI: 1.077-2.268; *P*=0.019), and fewer GDM prescriptions (HR: 1.876, 95% CI: 1.300-2.710; *P*=0.001) were independent predictors of one-year total mortality in patients with concomitant HFrEF and CKD (all *P*<0.05) (Table 2).

Multivariate analysis of all relevant variables presented in Supplementary Table 1 (the same as those variables included for total mortality analysis) indicated that advanced HF at discharge (HR: 1.624, 95% CI: 1.061-2.486; *P*=0.025), DBP at discharge (per mmHg decrement) (HR: 1.027, 95% CI: 1.008-1.045; *P*=0.004), LVEF (per % decrement) (HR: 1.028, 95% CI: 1.002-1.054; *P*=0.036), and fewer GDM prescriptions (HR: 1.859, 95% CI: 1.195-2.899; *P*=0.006) were independent predictors of one-year CV mortality in patients with HFrEF and CKD (all *P*<0.05) (Table 3).

### Impact of more and fewer GDM prescriptions on one-year total mortality and CV mortality in patients with concomitant HFrEF and CKD before and after PSM

Six hundred and twenty-five patients with HFrEF and CKD (314 patients with more GDM prescriptions and 311 patients with fewer GDM prescriptions) who had complete data, including demographics, comorbidities, echocardiographic parameters, vital signs at discharge, advanced HF at discharge, and laboratory data were included for PSM analysis (total 30 items in Table 4). After 1:1 PSM, there were 197 patients with more GDM prescriptions and 197 patients with fewer GDM prescriptions were included for further analysis (Fig. 1).

Table 4 shows the baseline characteristics of patients with HFrEF and CKD who received more and fewer GDM prescriptions before and after PSM. Before matching, patients with fewer GDM prescriptions were older, had lower BMI, and were more likely to be female. Additionally, the prevalence of several comorbidities, such as DM, advanced CKD, ICM, acute decompensation of chronic HF, PAOD, COPD, thyroid disorder, and cancer, was higher in patients with fewer GDM prescriptions than in those with more GDM prescriptions. Furthermore, patients with fewer GDM prescriptions had higher LVEF, lower hemoglobin levels, more advanced HF at discharge, and lower SBP and DBP at discharge. After matching, a group balance of baseline characteristics was achieved.

**Figure 3:**
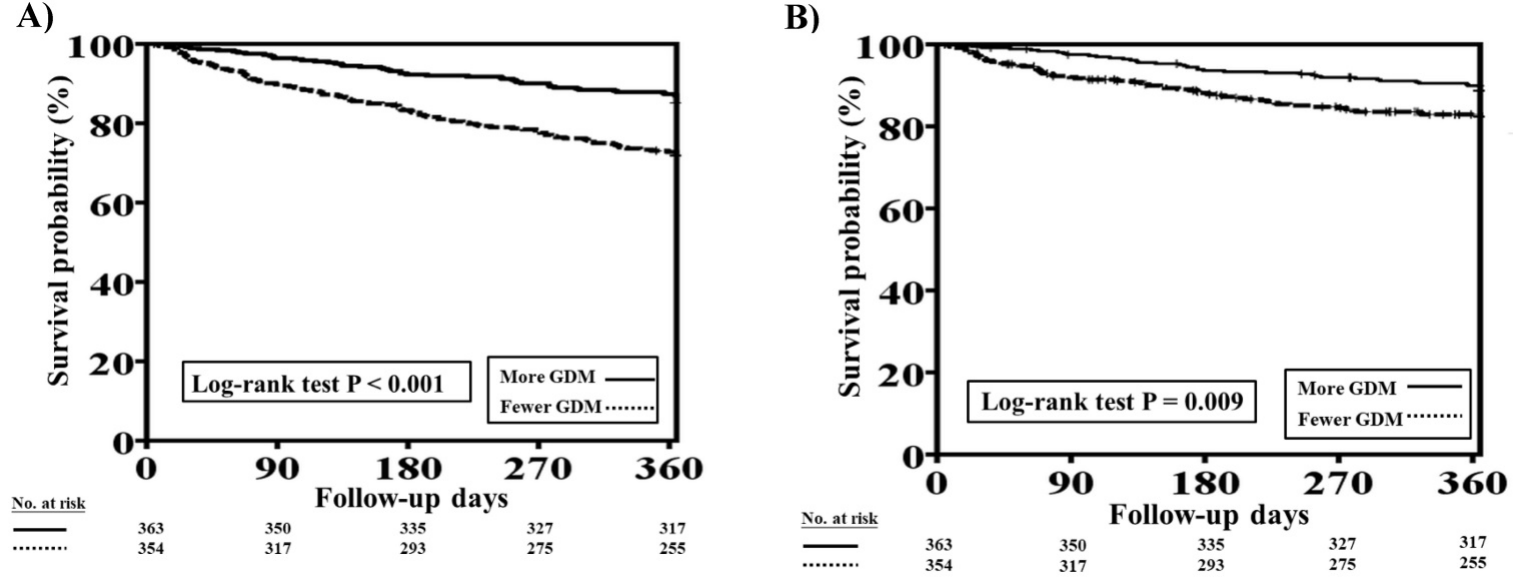
One-year total mortality and CV mortality rates in patients with concomitant HFrEF and CKD who had more and fewer GDM prescriptions. **A,** The Kaplan-Meier curve indicated that the one-year total mortality rate was higher in patients with concomitant HFrEF and CKD who received fewer GDM prescriptions than in those who had more GDM prescriptions (log-rank test, *P*<0.001). **B,** The Kaplan-Meier curve indicated that the one-year CV mortality rate was higher in patients with concomitant HFrEF and CKD who received fewer GDM prescriptions than in. those who had more GDM prescriptions (log-rank test, *P*=0.009). CKD, chronic kidney disease; CV, cardiovascular; GDM, guideline-directed medication; HFrEF, heart failure with reduced ejection fraction.

The one-year total mortality and CV mortality rates before PSM in patients with concomitant HFrEF and CKD who received more and fewer GDM prescriptions are presented in Figure 3A and 3B, respectively. Patients with fewer GDM prescriptions had higher one-year total mortality and CV mortality rates than those with more GDM prescriptions (log-rank test, *P*<0.001 for total mortality and *P*=0.009 for CV mortality).

**Figure 4:**
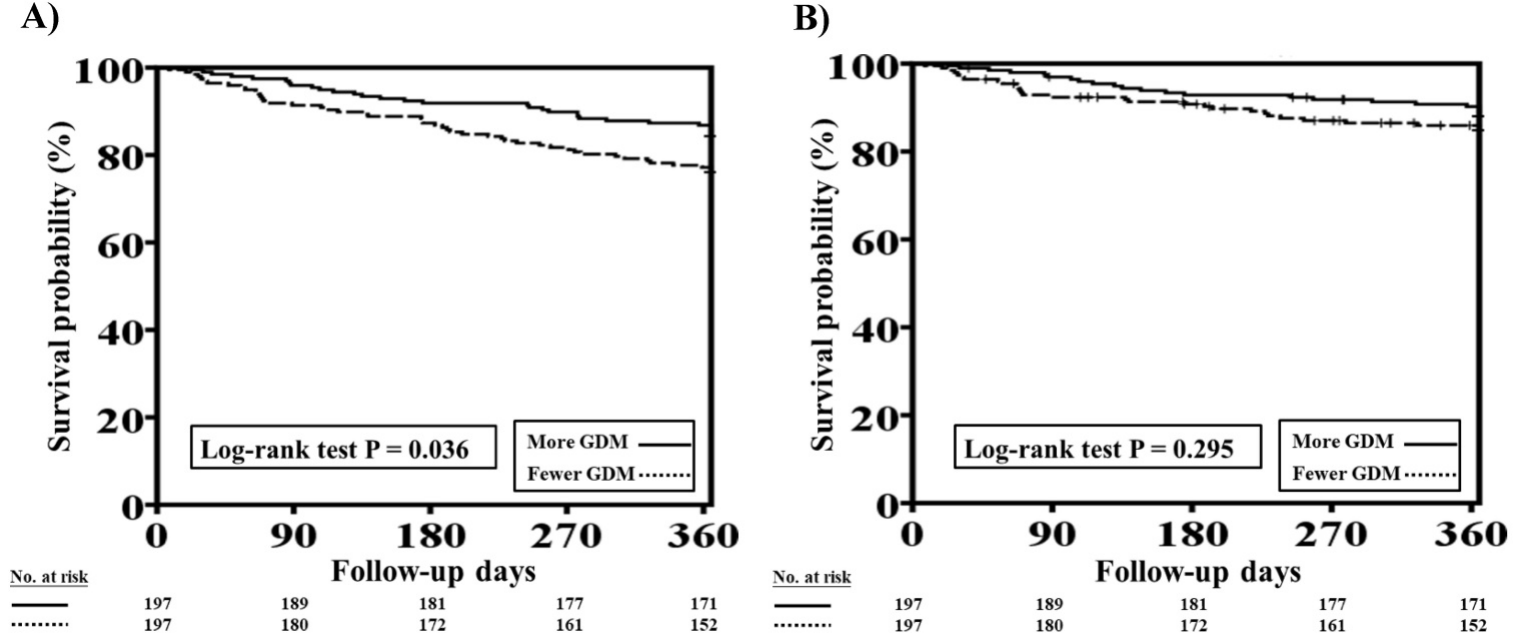
One-year total mortality and CV mortality rates in patients with concomitant HFrEF and CKD who had more and fewer GDM prescriptions after PSM. A, The Kaplan-Meier curve indicated that after PSM, the one-year mortality rate was higher in patients with concomitant HFrEF and CKD who received fewer GDM prescriptions than in those who had more GDM prescriptions (log-rank test, *P*=0.036). B, The Kaplan-Meier curve indicated that there were no significant differences in one-year CV mortality rate between patients with concomitant HFrEF and CKD who had fewer and more GDM prescriptions (log-rank test, *P*=0.295). CKD, chronic kidney disease; CV, cardiovascular; GDM, guideline-directed medication; HFrEF, heart failure with reduced ejection fraction; PSM, propensity score matching. Cox proportional hazard analysis showed the fewer GDMs were associated with a higher rate of total mortality (HR: 1.609, 95% CI: 1.021-2.535; P=0.040) as compared to more GDMs after adjusting all covariates used to generate PSM. The association regarding CV mortality was not significant (P=0.323).

The one-year total mortality and CV mortality rates after PSM in patients with HFrEF and CKD who received more and fewer GDM prescriptions are presented in Figure 4A and 4B, respectively. Patients with fewer GDM prescriptions still had a higher one-year total mortality rate than those with more GDM prescriptions (log-rank test, *P*=0.036 for total mortality and *P*=0.295 for CV mortality).

Cox proportional hazard analysis showed the fewer GDMs were associated with a higher rate of total mortality (HR: 1.609, 95% CI: 1.021-2.535; P=0.040) as compared to more GDMs after adjusting all covariates used to generate PSM. The association regarding CV mortality was not significant (P=0.323).

## Discussion

The propensity-matched analysis of HFrEF patients with CKD in the present study indicated that compared to fewer GDM prescriptions, more GDM prescriptions were associated with better clinical outcomes (one-year total mortality). Furthermore, this study showed that patients with advanced CKD had the lowest GDM prescription rate. Fewer GDM prescriptions, PAOD, thyroid disorder, advance HF at discharge, lower DBP at discharge, and digoxin use could predict one-year total mortality in patients with concomitant HFrEF and CKD. Fewer GDM prescriptions, lower LVEF, advanced HF at discharge, and lower DBP at discharge could predict one-year CV mortality in patients with concomitant HFrEF and CKD.

CKD is common and present in 30-50% of patients with HFrEF 9-11. All-cause mortality has been reported to be higher in HF patients with moderate to advanced CKD (HR: approximately 1.2-2.9) than in those without CKD.^9^-^11^ While the strategies for HF treatment are the same for patients with or without CKD, the presence of CKD raises special considerations with regard to GDM prescriptions, particularly for patients with eGFR <30 mL/min/1.73 m^2^ who have largely been excluded from clinical trials and in whom pharmacotherapy-related effects (hypovolemia, electrolyte imbalance, and hypotension) may considerably complicate therapy 12, 13. Dynamic changes in renal function during HF management have also been recognized to be poor prognostic factors 14, 15. However, a more precise definition and approach would be to combine a clinical response with changes in renal function measures to distinguish the pathophysiologically plausible entity 16.

A previous study showed that patients with renal dysfunction were less likely to receive important guideline-recommended therapies.^17^ Nonetheless, other studies reported that there were sustained benefits with GDM use in patients with worsening renal function (WRF) 18-20. In this HFrEF cohort study, we observed that HFrEF patients with advanced CKD had the lowest number of GDM prescriptions, followed by patients with moderate CKD, whereas patients without CKD had the highest number of GDM prescriptions. In patients with concomitant HFrEF and CKD, fewer GDM prescriptions were related to higher one-year mortality and CV mortality. In our study, fewer GDM prescriptions independently predicted one-year mortality and CV mortality. We found that even after adjustment for clinical comorbidities and confounding factors, patients with HFrEF and CKD who received more GDM prescriptions had better outcomes than those who received fewer GDM prescriptions. Therefore, our study suggests that the use of disease-modifying drugs was associated with better clinical outcomes in HFrEF patients with CKD at discharge. A position paper from the European Society of Cardiology suggests that careful interpretation of changes in renal function within an appropriate clinical context aids in determining further treatment strategies 21. Previous studies reported that the eGFR based on serum creatinine and cystatin C improved the prediction of 10-year HF risk in a large community population 22 and that a good diuretic response during acute HF management (an example of tubular function assessment) was associated with better clinical outcomes 23. Hence, it is clear that evaluation of renal function during acute HF should employ multiparameter-based analysis of decongestion, dynamic biomarker assessment, and clinical and technical assessment to constitute the best contemporary strategy.

Recent studies have shown that WRF was associated with increased mortality, but only when the HF status deteriorated 23-25. A meta-analysis of renin-angiotensin-aldosterone system (RAAS) inhibitors in systolic HF trials revealed that RAAS inhibitors confer greater benefit to participants with WRF than to those with no WRF 26. The new term “pseudo-WRF” refers to a patient with improved clinical status but increased serum creatinine levels 16. Patients with pseudo-WRF that occurs in the setting of complete decongestion have better outcomes than patients with WRF who did not have other trigger factors. Several studies have verified that if WRF occurs with the initiation of GDM prescriptions (ACEIs, ARBs, and MRAs), the beneficial effect of GDM therapies is maintained 26-28. The WRF induced by these RAAS inhibitors is not always associated with poor outcomes. The prescription of beta-blockers to HF patients with CKD has an even larger survival benefit according to the sub-analysis of several beta-blocker trials 29-31. The TSOC-HFrEF Registry defined CKD as patients at discharge, which may include patients with pseudo-WRF. The GDM prescription rate at discharge in the TSOC-HFrEF Registry is relatively low (ACEIs/ARBs, 62.1%; beta-blockers, 59.6%; and MRAs, 49.0%) 6. Consequently, a post-acute care program with a multidisciplinary team approach was launched to improve GDM prescriptions in the hope that clinical outcomes could be improved 32, 33.

A previous risk score prediction model developmental study in the MAGGIC meta-analysis identified lower LVEF, NYHA functional class, lower SBP, and serum creatinine levels as among the 13 predictors of mortality in HF patients, including those with preserved and reduced LVEF 34 The present study, which focused on patients with concomitant HFrEF and CKD, confirmed that both lower LVEF and advanced HF (NYHA functional class ≥3) could predict worse clinical outcomes. Furthermore, in our present study, lower DBP rather than lower SBP was associated with higher mortality. Lower SBP and DBP may limit GDM prescriptions for HF patients, and a lower blood pressure may also result in reduced coronary perfusion pressure, leading to a decreased myocardial oxygen supply, greater risk of myocardial ischemia and infarction, and subsequently worse CV outcomes 35. Additionally, CKD-related atherosclerosis may tend to simultaneously increase SBP and decrease DBP, resulting in a widened pulse pressure, which paves the way for CVD morbidity 36, 37. All of these factors may elucidate why DBP rather than SBP could predict worse outcomes in our cohort of patients with concomitant HFrEF and CKD.

Our study confirmed previous findings that non-cardiac comorbidities such as PAOD and thyroid disorder were associated with an increased mortality risk in patients with HFrEF 38. As 85% of digoxin is excreted by the kidneys, the risk of toxicity with this drug is very high among individuals with CKD 39. A previous cohort study reported that digoxin use was associated with a 28% increased mortality risk, which was related to increased serum digoxin concentrations and hypokalemia 40. Hence, considering the narrow therapeutic window, long half-life, and potential risk of lethal arrhythmias, most nephrologists generally avoid the use of digoxin for patients with advanced CKD and end-stage renal disease 41.

## Study limitations

The present study has some limitations. First, the effects of ARNi and ivabradine on patients with concomitant HFrEF and CKD were not analyzed because these drugs were not approved for use in Taiwan during the TSOC-HFrEF Registry period. Nevertheless, according to current clinical guidelines, ARNi and ivabradine are approved for use in patients with stable chronic HF 1, 2. Although the PIONEER-HF (Comparison of Sacubitril/Valsartan Versus Enalapril on Effect on NT-proBNP in Patients Stabilized from an Acute Heart Failure Episode) trial showed that ARNi can be safely initiated during admission and is associated with a reduction in cardiac biomarker and HF rehospitalization 42, we need to carefully interpret this result because the primary endpoint of the PIONEER-HF trial was not related to clinical outcomes. The PARADIGM-HF (Prospective Comparison of ARNi with ACEI to Determine Impact on Global Mortality and Morbidity in Heart Failure) trial also revealed that the effects of ARNi on reducing CV mortality or HF hospitalization were not modified by eGFR, even in patients with more advanced CKD 43. Second, while prescribed GDMs have been reported by previous studies to confer sustained benefits in HFrEF patients with moderate CKD, future HF intervention trials focusing on prespecified subgroups with advanced CKD and end-stage renal disease are required. Third, those patients with end-stage renal disease and HFrEF belong to a specific group and may have different clinical outcomes. However, we cannot have this information in this present study because the data regarding end-stage renal disease and dialysis was not included in this registry.

## Conclusion

CKD at discharge from HF hospitalization was associated with fewer GDM prescriptions, particularly in patients with more advanced CKD. The propensity-matched analysis revealed that the neurohormonal blockade effects by GDMs still confer survival benefit in patients with concomitant HFrEF and CKD. Therefore, a better understanding of the underlying cardiorenal physiology during acute HF admission may improve the initiation or continuation of GDM prescriptions.

## Supplementary Material

Supplementary tables.

Acknowledgements for 21 medical centers.

## Figures and Tables

**Table 1:** Baseline characteristics of discharged heart failure with reduced ejection fraction patients with and without advanced chronic kidney disease (n=1275)

Variables	Advanced CKD* (n=249)	Moderate CKD* (n=468)	Control* (n=558)	P value
**Demographics**				
Age (years)	68.5 ± 13.3	66.9 ± 14.7	57.5 ± 16.5^a,b^	<0.001
**Sex**				<0.001
Male	147 (59.0%)	335 (71.6%)^a^	440 (78.9%)^a,b^	
Female	102 (41.0%)	133 (28.4%)	118 (21.1%)	
Smoking	107 (43.0%)	230 (49.1%)	306 (54.8%)^a^	0.006
Alcoholism	2 (0.8%)	13 (2.8%)	27 (4.8%)^a^	0.009
**Comorbidities**				
BMI (kg/m^2^)	24.2 ± 4.6	25.6 ± 5.2^a^	25.5 ± 5.1^a^	0.001
AF	65 (26.1%)	133 (28.4%)	143 (25.6%)	0.584
HTN	85 (34.1%)	180 (38.5%)	166 (29.7%)^b^	0.013
DM	153 (61.4%)	217 (46.4%)^a^	193 (34.6%)^a,b^	<0.001
Dyslipidemia	60 (24.1%)	115 (24.6%)	115 (20.6%)	0.273
Old stroke	27 (10.8%)	54 (11.5%)	39 (7.0%)^b^	0.031
Old MI	74 (29.7%)	121 (25.9%)	122 (21.9%)^a^	0.048
PAOD	38 (15.3%)	31 (6.6%)^a^	15 (2.7%)^a,b^	<0.001
COPD	27 (10.8%)	50 (10.7%)	55 (9.9%)	0.875
OSA	6 (2.4%)	13 (2.8%)	17 (3.0%)	0.878
Thyroid disorder	11 (4.4%)	27 (5.8%)	22 (3.9%)	0.377
Hepatitis	21 (8.4%)	26 (5.6%)	32 (5.7%)	0.262
Depression	8 (3.2%)	7 (1.5%)	7 (1.3%)	0.127
Cancer	10 (4.0%)	11 (2.4%)	16 (2.9%)	0.448
Previous Valvular surgery	17 (6.8%)	22 (4.7%)	21 (3.8%)	0.165
***HF type***				0.028
New-onset HF	135 (54.2%)	196 (41.9%)	203 (36.4%)^a^	
Decompensated HF	114 (45.8%)	272 (58.1%)	355 (53.6%)	
***HF etiology***				<0.001
ICM	129 (51.8%)	204 (43.6%)^a^	201 (36.0%)^a,b^	
NICM	120 (48.2%)	264 (56.4%)	357 (64.0%)	
**Echocardiographic data†**				
LA size (mm)	45.9 ± 9.1	46.4 ± 8.6	46.4 ± 8.7	0.725
LVEF (%)	30.4 ± 8.1	28.5 ± 9.2^a^	27.7 ± 8.5^a^	<0.001
**Laboratory data†**				
BUN (mg/dl)	61.3 ± 31.5	29.8 ± 12.6^a^	18.8 ± 7.1^a,b^	<0.001
Cr (mg/dl)	4.6 ± 2.7	1.5 ± 0.3^a^	1.0 ± 0.2^a,b^	<0.001
eGFR (ml/min/1.73m^2^)	16.5 ± 8.1	46.1 ± 8.4^a^	86.4 ± 34.4^a,b^	<0.001
Na (meq/l)	136.3 ± 5.6	137.6 ± 4.4^a^	138.4 ± 3.9^a,b^	<0.001
K (meq/l)	4.3 ± 0.8	4.0 ± 0.6^a^	3.9 ± 0.5^a,b^	<0.001
Hgb (gm/dl)	10.9 ± 2.1	12.9 ± 2.3^a^	13.9 ± 2.0^a,b^	<0.001
**Vital signs and HF status at discharge**				
HR (beats/min)	80.2 ± 14.4	79.3 ± 14.3	81.6 ± 15.3^b^	0.042
SBP (mmHg)	125.9 ± 19.0	119.6 ± 18.6^a^	116.3 ± 17.3^a,b^	<0.001
DBP (mmHg)	71.0 ± 12.3	71.2 ± 13.6	72.5 ± 12.2	0.153
***NYHA functional class at discharge***				0.004
≤ II	167 (67.1%)	325 (69.4%)^a^	429 (76.9%)^a,b^	
≥ III	82 (32.9%)	143 (30.6%)	129 (23.1%)	
**Medication at discharge**				
ACEIs/ARBs	91 (36.5%)	287 (61.3%)^a^	400 (71.7%)^a,b^	<0.001
Beta-blocker	132 (53.0%)	285 (60.9%)	344 (61.6%)	0.055
Aldactone/Eplerenone	48 (19.3%)	229 (48.9%)^a^	324 (58.1%)^a,b^	<0.001
Diuretics	167 (67.1%)	361 (77.1%)^a^	408 (73.1%)	0.014
CCB	53 (21.3%)	54 (11.5%)^a^	45 (8.1%)^a^	<0.001
Digoxin	45 (18.1%)	126 (26.9%)^a^	163 (29.2%)^a^	0.004
Antiplatelet	174 (69.9%)	264 (56.4%)^a^	312 (55.9%)^a^	<0.001
Anticoagulation	34 (13.7%)	119 (25.4%)^a^	125 (22.4%)^a^	0.001
Anti-arrhythmia	30 (12.0%)	84 (17.9%)	84 (15.1%)	0.106
***Number of GDM prescription***	1.1 ± 0.8	1.7 ± 0.9^a^	1.9 ± 0.9^a,b^	<0.001
0	61 (24.5%)	42 (9.0%)	45 (8.1%)	
1	113 (45.4%)	138 (29.5%)	114 (20.4%)	
2	67 (26.9%)	201 (42.9%)	244 (43.7%)	
3	8 (3.2%)	87 (18.6%)	155 (27.8%)	
**Outcomes**				
**One-year mortality during follow-up**	63 (25.3%)	90 (19.2%)	61 (10.9%)^a,b^	<0.001
CV	39 (15.7%)	61 (13.0%)	42 (7.5%)^a,b^	0.001
Non-CV	24 (9.6%)	29 (6.2%)	20 (3.6%)^a^	0.002

Data are expressed as means ± SD or n (%). ACEIs/ARBs: angiotensin-converting enzyme inhibitors/angiotensin-receptor blockers; AF: atrial fibrillation; BMI: body mass index; BUN: blood urine nitrogen; CAD: coronary artery disease; CCB: calcium channel blocker; CKD: chronic kidney disease; COPD: chronic obstructive pulmonary disease; Cr: creatinine; CV: cardiovascular; DBP: diastolic blood pressure; DM: diabetes mellitus; eGFR: estimated glomerular filtration rate; ICM: ischemic cardiomyopathy; GDM: guideline-directed medication; HF: heart failure; Hgb: hemoglobin; HR: heart rate; HTN: hypertension; K: potassium; LA: left atrium; LVEF: left ventricular ejection fraction; MI: myocardial infarction; Na: sodium; NICM: non-ischemic cardiomyopathy; NYHA: New York Heart Association; OSA: obstructive sleep apnea; PAOD: peripheral artery occlusion disease; SBP: systolic blood pressure; SHF: systolic heart failure. *moderate CKD: 30 ml/min/1.73 m^2^ ≤ estimated glomerular filtration rate < 60 ml/min/1.73 m^2^; advanced CKD: estimated glomerular filtration rate < 30 ml/min/1.73 m^2^; control: estimated glomerular filtration rate ≥ 60 ml/min/1.73 m^2^. † Data first collected during the index hospitalization. ^a^P < 0.05 vs. advanced CKD. ^b^P < 0.05 vs. moderate CKD.

**Table 2:** Multivariate analysis for predictors of one-year total mortality in patients with HFrEF and CKD (N=717)

Variables	HR (95% CI)	*P* value
PAOD	1.828 (1.130-2.959)	0.014
Thyroid disorder	1.969 (1.101-3.521)	0.022
Advanced HF at discharge*	1.688 (1.192-2.391)	0.003
DBP at discharge (per mmHg decrement)	1.018 (1.004-1.033)	0.014
Digoxin use	1.563 (1.077-2.268)	0.019
Fewer GDM prescriptions^†^	1.876 (1.300-2.710	0.001

HFrEF: heart failure and a reduced ejection fraction; CKD: chronic kidney disease; CI: confidence interval; DBP: diastolic blood pressure, GDM: guideline-directed medication; HF: heart failure; HR: hazard ratio; NYHA: New York Heart Association; PAOD: peripheral artery occlusive disease. *Advanced HF was defined as HF, New York Heart Association functional class ≥3. †Few GDM prescriptions was defined as <2 GDM prescriptions. Variables including in the model: demographics (age, sex, body mass index), comorbidity (atrial fibrillation, diabetes mellitus, advanced chronic kidney disease, PAOD, chronic obstructive pulmonary disease, thyroid disorder, previous valvular surgery, HF type, HF etiology, advanced HF), laboratory and echocardiographic data (sodium, potassium, hemoglobin, left ventricular ejection fraction), vital signs at discharge (DBP), therapy (digoxin use, Few GDM prescriptions).

**Table 3:** Multivariate analysis for predictors of one-year cardiovascular death in patients with heart failure and a reduced ejection fraction and chronic kidney disease (N=717)

Variables	HR (95% CI)	*P* value
Advanced HF at discharge*	1.624 (1.061-2.486)	0.025
DBP at discharge (per mmHg decrement)	1.027 (1.008-1.045)	0.004
Left ventricular EF (per % decrement)	1.028 (1.002-1.054)	0.036
Fewer GDM prescriptions†	1.859 (1.195-2.899)	0.006

CI: confidence interval; DBP: diastolic blood pressure; EF: ejection fraction; GDM: guideline-directed medication; HF: heart failure; HR: hazard ratio. *Advanced HF was defined as HF, New York Heart Association functional class ≥3. †Few GDM prescriptions was defined as <2 GDM prescriptions. Variables included in the model: demographics (age, sex, body mass index), comorbidity (atrial fibrillation, diabetes mellitus, advanced chronic kidney disease, PAOD, chronic obstructive pulmonary disease, thyroid disorder, previous valvular surgery, HF type, HF etiology, advanced HF), laboratory and echocardiographic data (sodium, potassium, hemoglobin, left ventricular ejection fraction), vital signs at discharge (DBP), therapy (digoxin use, few GDM prescriptions).

**Table 4:** Baseline characteristics of heart failure with a reduced ejection fraction and chronic kidney disease patients with more and fewer guideline-direct medication prescriptions before and after propensity score matching

Variables	Before matching			After matching		
	More GDM* (n =314)	Fewer GDM* (n=311)	SMD	More GDM* (n =197)	Fewer GDM* (n=197)	SMD
Age (years)	64.6 ± 15.0	70.0 ± 13.2	0.388	67.7 ± 14.2	68.3 ± 13.5	0.043
Male sex	227 (72.3)	199 (64.0)	0.179	131 (66.5)	130 (66.0)	0.011
Smoking	160 (51.0)	135 (43.4)	0.152	92 (46.7)	88 (44.7)	0.041
Alcoholism	10 (3.2)	2 (0.6)	0.186	1 (0.5)	2(1.0)	0.058
BMI (kg/m^2^)	26.2 ± 5.4	24.1 ± 4.5	0.433	25.3±4.5	25.0 ± 4.6	0.066
Advanced CKD	66 (21.0)	152 (48.9)	0.611	64 (32.5)	59 (29.9)	0.055
AF	89 (28.3)	80 (25.7)	0.059	50 (25.4)	51 (25.9)	0.012
HTN	117 (37.3)	117 (37.6)	0.007	75 (38.1)	74 (37.6)	0.010
DM	151 (48.1)	171 (55.0)	0.138	101 (51.3)	95 (48.2)	0.061
Dyslipidemia	75 (23.9)	82 (26.4)	0.057	56 (28.4)	55 (27.9)	0.011
Old stroke	33 (10.5)	41 (13.2)	0.083	53 (11.7)	22 (11.2)	0.016
Old MI	71 (22.6)	97 (31.2)	0.194	54 (27.4)	46 (23.4)	0.093
ICM	133 (42.4)	160 (51.4)	0.183	97 (49.2)	90 (45.7)	0.071
Admitted due to decompensated HF	126 (40.1)	142 (45.7)	0.112	87 (44.2)	86 (43.7)	0.010
PAOD	24 (7.6)	39 (12.5)	0.163	18 (9.1)	18 (9.1)	0.001
COPD	27 (8.6)	41 (13.2)	0.148	21 (10.7)	19 (9.6)	0.034
OSA	12 (3.8)	6 (1.9)	0.113	5 (2.5)	6 (3.0)	0.031
Thyroid disorder	11 (3.5)	20 (6.4)	0.135	9 (4.6)	9 (4.6)	<0.001
Hepatitis	19 (6.1)	24 (7.7)	0.066	15 (7.6)	10 (5.1)	0.104
Depression	5 (1.6)	9 (2.9)	0.088	5 (2.5)	5 (2.5)	<0.001
Cancer	5 (1.6)	10 (3.2)	0.106	5 (2.5)	5 (2.5)	<0.001
Previous valvular surgery	18 (5.7)	15 (4.8)	0.041	10 (5.1)	9 (4.6)	0.024
**Echocardiographic data†**						
LVEF (%)	27.9 ± 9.1	29.9 ± 8.1	0.236	29.2 ± 9.2	29.0 ± 8.1	0.021
**Vital signs at discharge**						
HR (beats per minute)	92.9 ± 23.7	93.7 ± 21.1	0.037	92.5 ± 24.4	92.7 ± 20.6	0.007
SBP (mmHg)	135.3 ± 32.4	130.4 ± 26.9	0.162	133.5 ± 31.4	131.2 ± 27.3	0.077
DBP (mmHg)	83.1 ± 21.1	76.2 ± 18.1	0.348	80.7 ± 20.3	78.7 ± 19.1	0.101
Advanced HF at discharge	90 (28.7)	109 (35.0)	0.137	67 (34.0)	66 (33.5)	0.011
**Laboratory data†**						
Na (meq/l)	137.3 ± 4.7	136.8 ± 4.9	0.100	136.9 ± 4.9	137.3 ± 4.3	0.071
K (meq/l)	4.1 ± 0.7	4.2 ± 0.7	0.093	4.1 ± 0.7	4.1 ± 0.7	0.010
Hgb (gm/dl)	12.8 ± 2.3	11.6 ± 2.5	0.504	12.3 ± 2.2	12.1 ± 2.5	0.088

Data was expressed as n (%) or mean ± standard deviation. AF: atrial fibrillation; BMI: body mass index; CAD: coronary artery disease; COPD: chronic obstructive pulmonary disease; DBP: diastolic blood pressure; DM: diabetes mellitus; GDM: guideline-directed medication; HF: heart failure; HR: heart rate; HTN: hypertension; LA: left atrium; LVEF: left ventricular ejection fraction; MI: myocardial infarction; OSA: obstructive sleep apnea; PAOD: peripheral artery occlusion disease; SBP: systolic blood pressure. *More GDM prescriptions: GDM prescriptions ≥ 2; Fewer GDM prescriptions: GDM prescriptions < 2. † Data collected during index hospitalization.
