# Supplementary material for: Feature and impact of guideline-directed medication prescriptions for heart failure with reduced ejection fraction accompanied by chronic kidney disease
Source: Int J Med Sci. 2021 Apr 28;18(12):2570–80. doi: 10.7150/ijms.55119 (PMC8176167; doi:10.7150/ijms.55119)
Supplement: Supplementary file 1 — Supplementary tables. [file ijmsv18p2570s1.pdf]

Supplemental table 1. Baseline characteristics of discharged heart failure with reduced ejection fraction and chronic kidney patients with and without one-year total mortality (n=717)

| Variables | Mortality (n=153) | Alive (n=564) | P value | HR (95% CI) |
|---|---|---|---|---|
| **Demographics** | | | | |
| Age | 70.3 ± 12.1 | 66.7 ± 14.7 | 0.008 | 1.016 (1.004-1.028) |
| Men | 98 (64.1%) | 384 (68.1%) | 0.422 | 0.873 (0.628-1.215) |
| Smoking | 68 (44.4%) | 269 (47.7%) | 0.502 | 0.897 (0.652-1.233) |
| Alcoholism | 1 (0.7%) | 14 (2.5%) | 0.210 | 0.284 (0.040-2.031) |
| BMI | 24.0 ± 4.9 | 25.5 ± 5.0 | 0.001 | 0.939 (0.904-0.975) |
| **Comorbidities** | | | | |
| AF | 48 (31.4%) | 150 (26.6%) | 0.302 | 1.197 (0.851-1.684) |
| HTN | 49 (32.0%) | 216 (38.3%) | 0.156 | 0.782 (0.557-1.098) |
| DM | 91 (59.5%) | 279 (49.5%) | 0.038 | 1.408 (1.019-1.944) |
| Dyslipidemia | 39 (25.5%) | 136 (24.1%) | 0.688 | 1.077 (0.749-1.550) |
| Old stroke | 22 (14.4%) | 59 (10.5%) | 0.201 | 1.343 (0.855-2.109) |
| Old MI | 42 (27.5%) | 153 (27.1%) | 0.902 | 1.022 (0.717-1.458) |
| Advanced CKD | 63 (41.2%) | 186 (33.0%) | 0.043 | 1.395 (1.011-1.924) |
| PAOD | 22 (14.4%) | 47 (8.3%) | 0.016 | 1.741 (1.108-2.735) |
| COPD | 24 (15.7%) | 53 (9.4%) | 0.031 | 1.618 |

| | | | | |
|---|---|---|---|---|
| | | | | (1.046-2.501) |
| OSA | 3 (2.0%) | 16 (2.8%) | 0.526 | 0.691 (0.220-2.167) |
| Thyroid disorder | 15 (9.8%) | 23 (4.1%) | 0.007 | 2.090 (1.226-3.561) |
| Cancer | 3 (2.0%) | 18 (3.2%) | 0.480 | 0.662 (0.211-2.076) |
| Hepatitis | 10 (6.5%) | 37 (6.6%) | 0.955 | 0.982 (0.517-1.864) |
| Depression | 4 (2.6%) | 11 (2.0%) | 0.631 | 1.275 (0.472-3.442) |
| Previous valvular surgery | 15 (9.8%) | 24 (4.3%) | 0.009 | 2.043 (1.199-3.482) |
| HF type | | | 0.359 | 1.160 (0.845-1.595) |
| New-onset HF | 71 (46.4%) | 239 (42.4%) | | |
| Decompensated HF | 82 (53.6%) | 325 (57.6%) | | |
| HF etiology | | | 0.774 | 1.048 (0.763-1.439) |
| ICM | 73 (47.7%) | 260 (46.1%) | | |
| NICM | 80 (52.3%) | 304 (53.9%) | | |
| **Echo data*** | | | | |
| LA size (mm) | 46.5 ± 8.9 | 46.1 ± 8.7 | 0.571 | 1.005 (0.987-1.024) |
| LVEF (%) | 28.2 ± 8.3 | 29.4 ± 9.0 | 0.134 | 0.986 (0.968-1.004) |
| **Laboratory data*** | | | | |
| BUN | 49.3 ± 28.4 | 38.7 ± 24.9 | <0.001 | 1.010 (1.006-1.015) |
| Cr | 2.8 ± 2.2 | 2.5 ± 2.2 | 0.110 | 1.153 (0.989-1.121) |
| eGFR | 32.8 ± 16.4 | 36.6 ± 16.3 | 0.008 | 0.987 (0.978-0.997) |
| Na | 136.5 ± 5.1 | 137.3 ± 4.8 | 0.074 | 0.972 |

| | | | | |
|---|---|---|---|---|
| | | | | (0.943-1.003) |
| K | 4.2 ± 0.7 | 4.1 ± 0.7 | 0.173 | 1.165 (0.935-1.451) |
| Hgb | 11.8 ± 2.4 | 12.3 ± 2.5 | 0.018 | 0.922 (0.862-0.986) |
| **Vital signs and HF status at discharge** | | | | |
| HR | 80.7 ± 15.2 | 79.3 ± 14.1 | 0.200 | 1.007 (0.996-1.018) |
| SBP | 119.3 ± 20.2 | 122.4 ± 18.6 | 0.060 | 0.992 (0.983-1.000) |
| DBP | 68.1 ± 12.8 | 72.0 ± 13.1 | 0.002 | 0.980 (0.967-0.993) |
| HF, NYHA Functional class at discharge | | | 0.003 | 1.624 (1.177-2.240) |
| I + II | 90 (58.8%) | 402 (71.2%) | | |
| III +IV | 63 (41.2%) | 162 (28.7%) | | |
| **Medication** | | | | |
| ACEI/ARB | 67 (43.8%) | 311 (55.1%) | 0.010 | 0.658 (0.478-0.905) |
| Beta-blocker | 41 (46.4%) | 346 (61.3%) | 0.001 | 0.750 (0.418-0.790) |
| Aldactone/eplerenone | 88 (41.1%) | 513 (48.4%) | 0.093 | 0.750 (0.536-1.049) |
| Diuretics | 105 (68.6%) | 423 (75.0%) | 0.099 | 0.750 (0.533-1.055) |
| CCB | 20 (13.1%) | 87 (15.4%) | 0.433 | 0.829 (0.518-1.326) |
| Digoxin | 49 (32.0%) | 122 (21.6%) | 0.012 | 1.546 (1.101-2.172) |
| Antiplatelet | 91 (59.5%) | 347 (61.5%) | 0.733 | 0.945 (0.685-1.305) |
| Anticoagulation | 31 (20.3%) | 122 (21.6%) | 0.664 | 0.916 (0.618-1.359) |

| | | | | |
|---|---|---|---|---|
| Anti-arrhythmia | 27 (17.6%) | 87 (15.4%) | 0.454 | 1.172 (0.773-1.776) |
| Number of GDM prescriptions! | 1.2 ± 0.9 | 1.6 ± 0.9 | <0.001 | 0.675 (0.564-0.809) |
| 0 | 33 (21.6%) | 70 (12.4%) | | |
| 1 | 66 (43.1%) | 185 (32.8%) | | |
| 2 | 39 (25.5%) | 229 (40.6%) | | |
| 3 | 15 (9.8%) | 80 (14.2%) | | |
| Number of GDM prescriptions ≥ 2 | 54 (35.3%) | 309 (54.8%) | <0.001 | 0.478 (0.343-0.666) |

Data are expressed as means ± SD or % (n).

ACEIs/ARBs= angiotensin-converting enzyme inhibitors/angiotensin-receptor blockers; AF= atrial fibrillation; BMI= body mass index; BUN= blood urine nitrogen; CAD= coronary artery disease; CCB= calcium channel blocker; CKD= chronic kidney disease; COPD= chronic obstructive pulmonary disease; Cr= creatinine; CV= cardiovascular; DBP= diastolic blood pressure; DM= diabetes mellitus; Echo= echocardiography; GDM= guideline-directed medication; HF= heart failure; Hgb= hemoglobin; HR= heart rate; HTN= hypertension; ICM= ischemic cardiomyopathy; K= potassium; LA= left atrium; LVEF= left ventricular ejection fraction; MI= myocardial infarction; Na= sodium; NICM= non-ischemic cardiomyopathy; OSA= obstructive sleep apnea; PAOD= peripheral artery occlusion disease; SBP= systolic blood pressure; SHF= systolic heart failure.

* Data first collected during the index hospitalization

Supplemental table 2. Baseline characteristics of discharged heart failure with reduced ejection fraction and chronic kidney patients with and without one-year CV mortality (n=717)

| Variables | Mortality (n=100) | Alive (n=617) | P value | HR (95% CI) |
|---|---|---|---|---|
| **Demographics** | | | | |
| Age | 69.5 ± 12.4 | 67.1 ± 14.5 | 0.106 | 1.012 (0.997-1.026) |
| Men | 69 (69.0%) | 413 (66.9%) | 0.680 | 1.093 (0.716-1.670) |
| Smoking | 47 (47.0%) | 290 (47.0%) | 0.983 | 0.996 (0.672-1.475) |
| Alcoholism | 1 (1.0%) | 14 (2.3%) | 0.414 | 0.440 (0.061-3.155) |
| BMI | 24.7 ± 5.4 | 25.2 ± 5.0 | 0.264 | 0.976 (0.935-1.019) |
| **Comorbidity** | | | | |
| AF | 29 (29.0%) | 169 (27.4%) | 0.767 | 1.068 (0.693-1.644) |
| HTN | 25 (25.0%) | 240 (38.9%) | 0.011 | 0.554 (0.352-0.871) |
| DM | 61 (61.0%) | 309 (50.1%) | 0.051 | 1.493 (0.999-2.231) |
| Dyslipidemia | 25 (25%) | 150 (24.3%) | 0.841 | 1.048 (0.666-1.647) |
| Old stroke | 11 (11.0%) | 70 (11.3%) | 0.964 | 0.986 (0.527-1.844) |
| Old MI | 25 (25.0%) | 170 (27.6%) | 0.654 | 0.902 (0.573-1.418) |
| Advanced CKD | 39 (39.0%) | 210 (34.0%) | 0.242 | 1.271 (0.850-1.899) |
| PAOD | 15 (15.0%) | 54 (8.8%) | 0.033 | 1.818 (1.050-3.148) |
| COPD | 12 (12.0%) | 65 (10.5%) | 0.585 | 1.183 (0.647-2.162) |
| OSA | 3 (3.0%) | 16 (2.6%) | 0.906 | 0.933 (0.296-2.943) |
| Thyroid disorder | 10 (10.0%) | 28 (4.5%) | 0.025 | 2.110 (1.098-4.056) |
| Cancer | 1 (1.0%) | 20 (3.2%) | 0.278 | 0.336 (0.047-2.411) |
| Hepatitis | 5 (5.0%) | 42 (6.8%) | 0.509 | 0.739 (0.301-1.816) |
| Depression | 3 (3.0%) | 12 (1.9%) | 0.515 | 1.464 (0.464-4.619) |
| Previous valvular surgery | 11 (11.0%) | 28 (4.5%) | 0.009 | 2.294 (1.226-4.292) |
| HF type | | | 0.390 | 1.188 (0.802-1.760) |
| New-onset HF | 47 (47.0%) | 263 (42.6%) | | |
| Decompensated HF | 53 (53.0%) | 354 (57.4%) | | |
| HF etiology | | | 0.624 | 1.103 (0.745-1.632) |
| ICM | 49 (49.0%) | 284 (46.0%) | | |
| NICM | 51 (51.0%) | 333 (54.0%) | | |

| | | | | |
|---|---|---|---|---|
| **Echo data*** | | | | |
| LA size (mm) | 47.0 ± 8.7 | 46.1 ± 8.8 | 0.325 | 1.011 (0.989-1.035) |
| LVEF (%) | 27.6 ± 8.1 | 29.4 ± 9.0 | 0.057 | 0.978 (0.956-1.001) |
| **Laboratory data*** | | | | |
| BUN | 47.5 ± 26.8 | 39.9 ± 25.8 | 0.004 | 1.009 (1.003-1.015) |
| Cr | 2.6 ± 2.1 | 2.6 ± 2.2 | 0.645 | 1.020 (0.936-1.112) |
| eGFR | 34.9 ± 16.3 | 35.9 ± 16.4 | 0.384 | 0.995 (0.983-1.007) |
| Na | 136.3 ± 5.5 | 137.3 ± 4.8 | 0.050 | 0.964 (0.929-1.000) |
| K | 4.2 ± 0.7 | 4.1 ± 0.7 | 0.425 | 1.118 (0.850-1.471) |
| Hgb | 11.9 ± 2.4 | 12.3 ± 2.5 | 0.191 | 0.947 (0.872-1.028) |
| **Vital signs and HF status at discharge** | | | | |
| HR | 81.1 ± 14.2 | 79.3 ± 14.4 | 0.184 | 1.009 (0.996-1.023) |
| SBP | 117.9 ± 21.0 | 122.4 ± 18.6 | 0.025 | 0.987 (0.977-0.998) |
| DBP | 67.2 ± 13.1 | 71.8 ± 13.0 | 0.002 | 0.974 (0.959-0.990) |
| NYHA functional class at discharge | | | 0.020 | 1.606 (1.078-2.392) |
| I + II | 59 (59.0%) | 433 (70.2%) | | |
| III + IV | 41 (41.0%) | 184 (29.8%) | | |
| **Medication** | | | | |
| ACEI/ARB | 45 (45.0%) | 333 (54.0%) | 0.069 | 0.694 (0.468-1.029) |
| Beta-blocker | 50 (50.0%) | 367 (59.5%) | 0.044 | 0.668 (0.451-0.988) |
| Aldactone/eplerenone | 35 (35.0%) | 242 (39.2%) | 0.315 | 0.810 (0.537-1.222) |
| Diuretics | 26 (26.0%) | 163 (26.4%) | 0.918 | 0.977 (0.625-1.527) |
| CCB | 13 (13.0%) | 94 (15.2%) | 0.515 | 0.824 (0.460-1.476) |
| Digoxin | 34 (34.0%) | 137 (22.2%) | 0.014 | 1.678 (1.109-2.538) |
| Antiplatelet | 63 (63.0%) | 375 (60.8%) | 0.657 | 1.096 (0.731-1.645) |
| Anticoagulation | 21 (21.0%) | 132 (21.4%) | 0.866 | 0.959 (0.593-1.552) |
| Anti-arrhythmia | 18 (18.0%) | 96 (15.6%) | 0.484 | 1.200 (0.720-1.998) |
| Number of GDM prescriptions | 1.3 ± 0.9 | 1.5 ± 0.9 | 0.007 | 0.737 (0.591-0.920) |
| 0 | 21 (21.0%) | 82 (13.3%) | | |
| 1 | 39 (39.0%) | 212 (34.4%) | | |
| 2 | 29 (29.0%) | 239 (38.7%) | | |

| | | | | |
|---|---|---|---|---|
| 3 | 11 (11.0%) | 84 (13.6%) | | |
| Number of GDM prescriptions ≥ 2 | 40 (40.0%) | 323 (52.4%) | 0.010 | 0.589 (0.395-0.879) |

Data are expressed as means ± SD or % (n).

ACEIs/ARBs= angiotensin-converting enzyme inhibitors/angiotensin-receptor blockers; AF= atrial fibrillation; BMI= body mass index; BUN= blood urine nitrogen; CAD= coronary artery disease; CCB= calcium channel blocker; CKD= chronic kidney disease; COPD= chronic obstructive pulmonary disease; Cr= creatinine; CV= cardiovascular; DBP= diastolic blood pressure; DM= diabetes mellitus; Echo= echocardiography; GDM= guideline-directed medication; HF= heart failure; Hgb= hemoglobin; HR= heart rate; HTN= hypertension; ICM= ischemic cardiomyopathy; K= potassium; LA= left atrium; LVEF= left ventricular ejection fraction; MI= myocardial infarction; Na= sodium; NICM= non-ischemic cardiomyopathy; OSA= obstructive sleep apnea; PAOD= peripheral artery occlusion disease; SBP= systolic blood pressure; SHF= systolic heart failure.

* Data first collected during the index hospitalization.
