# Supplementary material for: Feature and impact of guideline-directed medication prescriptions for heart failure with reduced ejection fraction accompanied by chronic kidney disease
Source: Int J Med Sci. 2021 Apr 28;18(12):2570–80. doi: 10.7150/ijms.55119 (PMC8176167; doi:10.7150/ijms.55119)
Supplement: Supplementary file 2 — Acknowledgements for 21 medical centers. [file ijmsv18p2570s2.pdf]

**The 21 medical centers that treated the patients enrolled in this study are listed below in alphabetical order**

Chang Gung Memorial Hospital, Keelung, Taiwan; Chang Gung Memorial Hospital, Linkou, Taiwan; Cheng Hsin General Hospital, Taipei, Taiwan; Chimei Medical Center, Tainan, Taiwan; China Medical University Hospital, Taichung, Taiwan; Chung-Shan Medical University Hospital, Taichung, Taiwan; E-Da Hospital, Kaohsiung, Taiwan; Far Eastern memorial Hospital, New Taipei City, Taiwan; Hualien Tzu Chi Hospital, Buddhist Tzu Chi Medical Foundation, Hualien, Taiwan; Kaohsiung Chang Gung Memorial Hospital, Kaohsiung, Taiwan; Kaohsiung Medical University Chung-Ho Memorial Hospital, Kaohsiung, Taiwan; Kaohsiung Veterans General Hospital, Kaohsiung, Taiwan; MacKay Memorial Hospital, Taipei, Taiwan; National Cheng Kung University Hospital, Tainan, Taiwan; National Taiwan University Hospital, Hsinchu Branch; National Taiwan University Hospital, Taipei, Taiwan; Shin Kong Wu Ho-Su Memorial Hospital, Taipei, Taiwan; Taichung Veterans General Hospital, Taichung, Taiwan; Taipei Tzu Chi Hospital, Buddhist Tzu Chi Medical Foundation, New Taipei City, Taiwan; Taipei Veterans General Hospital, Taipei, Taiwan; Tri-Service General Hospital and National Defense Medical Center, Taipei, Taiwan.
